# Impaired Bone Formation in *Pdia3* Deficient Mice

**DOI:** 10.1371/journal.pone.0112708

**Published:** 2014-11-18

**Authors:** Yun Wang, Alexandr Nizkorodov, Kelsie Riemenschneider, Christopher S. D. Lee, Rene Olivares-Navarrete, Zvi Schwartz, Barbara D. Boyan

**Affiliations:** 1 Wallace H. Coulter Department of Biomedical Engineering, Georgia Institute of Technology, Atlanta, Georgia, United States of America; 2 Department of Biomedical Engineering, Virginia Commonwealth University, Richmond, Virginia, United States of America; Inserm U606 and University Paris Diderot, France

## Abstract

1α,25-dihydroxyvitamin D_3_ [1α,25(OH)_2_D_3_] is crucial for normal skeletal development and bone homeostasis. Protein disulfide isomerase family A, member 3 (PDIA3) mediates 1α,25(OH)_2_D_3_ initiated-rapid membrane signaling in several cell types. To understand its role in regulating skeletal development, we generated *Pdia3*-deficient mice and examined the physiologic consequence of *Pdia3*-disruption in embryos and *Pdia3* ^+/−^ heterozygotes at different ages. No mice homozygous for the *Pdia3*-deletion were found at birth nor were there embryos after E12.5, indicating that targeted disruption of the *Pdia3* gene resulted in early embryonic lethality. *Pdia3*-deficiency also resulted in skeletal manifestations as revealed by µCT analysis of the tibias. In comparison to wild type mice, *Pdia3* heterozygous mice displayed expanded growth plates associated with decreased tether formation. Histomorphometry also showed that the hypertrophic zone in *Pdia3* ^+/−^ mice was more cellular than seen in wild type growth plates. Metaphyseal trabecular bone in *Pdia3* ^+/−^ mice exhibited an age-dependent phenotype with lower BV/TV and trabecular numbers, which was most pronounced at 15 weeks of age. Bone marrow cells from *Pdia3* ^+/−^ mice exhibited impaired osteoblastic differentiation, based on reduced expression of osteoblast markers and mineral deposition compared to cells from wild type animals. Collectively, our findings provide *in vivo* evidence that PDIA3 is essential for normal skeletal development. The fact that the *Pdia3* ^+/−^ heterozygous mice share a similar growth plate and bone phenotype to *nVdr* knockout mice, suggests that PDIA3-mediated rapid membrane signaling might be an alternative mechanism responsible for 1α,25(OH)_2_D_3_’s actions in regulating skeletal development.

## Introduction

The vitamin D metabolite 1α,25-dihydroxyvitamin D_3_ [1α,25(OH)_2_D_3_] is an essential regulator of skeletal development and homeostasis [1]. At the cellular level, it directly regulates proliferation and differentiation of growth plate chondrocytes and osteoblasts [2], [3]. 1α,25(OH)_2_D_3_ exerts its biological effects through two types of receptors: the traditional nuclear steroid hormone vitamin D receptor (nVDR) and a membrane-associated receptor, protein disulfide isomerase A3 (PDIA3), also referred to as 1α,25(OH)_2_D_3_-membrane associated rapid response steroid binding protein (1,25-MARRS), GRP58, ERp60, ERp58 and ERp57 [4], [5].

PDIA3 is a multiple function protein of the protein disulfide isomerase family [6]. It is well known as a chaperonin, which can assist glycoprotein folding and major histocompatibility complex I loading [7]. It regulates endoplasmic reticulum stress and controls cell survival [8]. It has also been shown to mediate 1α,25(OH)_2_D_3_ initiated rapid membrane responses in a variety of cell types. Nemere *et al*. have demonstrated that PDIA3 directly contributes to 1α,25(OH)_2_D_3_ induced calcium uptake by intestinal cells in chicks and rats, which is important for mineral ion homeostasis and bone mineral mass accumulation [9].

We have shown that PDIA3 is required for 1α,25(OH)_2_D_3_ activated protein kinase C-α (PKCα) signaling in both growth plate chondrocytes and osteoblasts [2], [3]. Upon binding to PDIA3 in caveolae on the membrane, 1α,25(OH)_2_D_3_ activates phospholipase A2 (PLA2) and PLC, leading to downstream activation of PKC [10]–[12] and extracellular regulated kinase (ERK) mitogen activated protein kinases 1 and 2 (ERK1/2) [2], [12], [13]. In addition to its non-genomic effects, PDIA3-dependent 1α,25(OH)_2_D_3_ rapid membrane signaling also leads to genomic regulation of several osteoblast markers and bone matrix mineralization in osteoblast cultures [12], [13]. These *in vitro* data suggest that the PDIA3-mediated rapid membrane signaling pathway may play an important role in 1α,25(OH)_2_D_3_ regulation of skeletal development *in vivo*.

Cell culture studies support this. Growth plate chondrocytes from *nVdr* knockout mice continue to exhibit increased PLA2, PLC and PKC activity in response to 1α,25(OH)_2_D_3_ [14]. The mechanism by which 1α,25(OH)_2_D_3_ elicits its actions in growth plate chondrocytes involves direct interaction with caveolin-1 (CAV-1) in caveolae microdomains [15]. Interestingly, growth plate chondrocytes from *Cav-1* knockout mice fail to respond to 1α,25(OH)_2_D_3_ with an increase in PKC [16]. Similarly, chondrocytes lacking intact caveolae also lack this rapid response to the vitamin D metabolite. Moreover, *Cav-1* ^−/−^ knockout mice exhibit a growth plate phenotype characterized by few hypertrophic chondrocytes compared to their *Cav-1* ^+/+^ littermates [15].

The skeletal actions of 1α,25(OH)_2_D_3_ have been studied in animal models and clinically. Vitamin D deficiency in animals and children results in rickets, characterized by inadequate calcification of the growth plate and adjacent metaphyseal bone [1], [17]. Several *nVdr* knockout mice models have been created to study the mechanisms involved in the development of the skeletal phenotype of hereditary vitamin D resistant rickets, a human disease associated with *nVdr* mutations [18]–[20]. These mice exhibit a rachitic growth plate phenotype after weaning, supporting the hypothesis that a functional VDR is required for bone development. Later studies showed that the rickets could be healed by a calcium-rich rescue diet, suggesting that the rachitic changes are a direct consequence of impaired endocrine function of 1α,25(OH)_2_D_3_ on mineral homeostasis, rather than impaired VDR-dependent genomic actions on bone [21].

Recent studies examining effects of the rescue diet on the morphology of the growth plate in *nVdr^−/−^* mice showed that although the rescue diet restored mineral deposition, it did not restore formation of mineralized tethers to normal levels [22]. Tethers are mineralized regions in the growth plate that link the epiphysis to the metaphysis, providing biomechanical stability during long bone growth. They are distributed in a distinctive pattern inside the circumference of normal growth plates as well as within the central region. *nVdr^−/−^* mice lack the circumferential tethers whether they are fed the rescue diet or not [22], indicating that their formation is VDR-dependent and is not secondary to 1α,25(OH)_2_D_3_’s actions on mineral ion homeostasis. Whether they are PDIA3-dependent or not is not known.

Although these *in vitro* and *in vivo* data collectively suggest an important role for PDIA3-mediated 1α,25(OH)_2_D_3_ rapid membrane signaling in skeletal development, its involvement in endochondral bone formation in vivo remains unknown. Attempts to generate a *Pdia3*-null mouse have met with frustration, as global ablation of the protein has resulted in embryonic lethality [7], [23]. Accordingly, mice that exhibited specific knockout in B-cells were established to assess the role of PDIA3 in immune function [7] and mice with specific knockout in intestinal epithelial cells were established to assess the role of PDIA3 in mineral ion transport [23]. In the present study, we opted to retain the global knockout approach and to examine heterozygous mice in order to gain a better understanding of how PDIA3 contributes to post-fetal musculoskeletal development, which involves multiple tissues and both systemic and biomechanical regulatory stimuli. Our results show that *Pdia3* ^+/−^ mice exhibit a mild skeletal phenotype characterized by enlarged growth plates, reduced tether and trabecular bone formation, and impaired osteogenic potential of calvarial osteoblasts in vitro.

## Materials and Methods

### Ethics statement

All animal work was conducted under the approval of the Georgia Institute of Technology IACUC.

### Generation of *Pdia3* knockout mice

**Figure 1:**
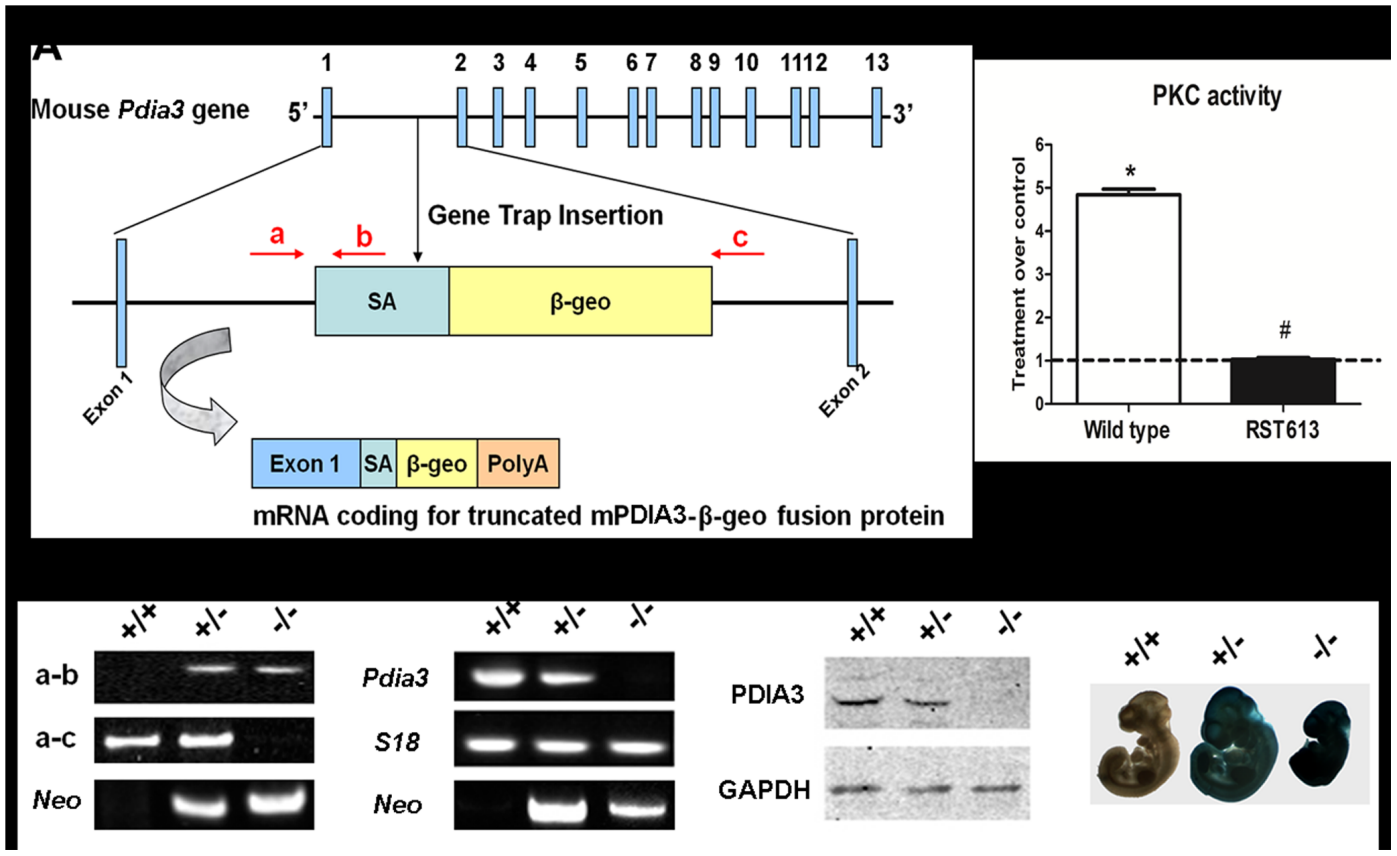
Generation of *Pdia3* knockout mouse. A schematic representation of ablation of *Pdia3* gene using gene trap vector inserted into the intron 1 of mouse *Pdia3* gene. The exons are numbered and indicated by solid blue boxes. Primers used for genotyping are indicated as a, b and c (A). PKC activity of 1,25(OH)_2_D_3_ treated wild type and RST613 cells (B). *P<0.05 vs. vehicle treatment, ^#^ *P*<0.05 vs. wild type. The samples collected from E10.5 embryos were verified using genotyping (C), RT-PCR (D), western blot (E), and X-gal staining (F).

A mouse embryonic stem cell (ESC) line (RST613, strain 129/OlaHsd) (BayGenomics, USA) with an insertional mutation in intron 1 of the *Pdia3* gene was created using a gene-trapping vector (Fig. 1A). The gene-trapped locus was predicted to yield a fusion transcript containing exon 1 of the *Pdia3* gene and a reporter gene, *βgeo* encoding a fusion protein of β-galactosidase and neomycin phosphotransferase II. The formation of non-functional fusion protein was verified by examining 1α,25(OH)_2_D_3_-induced PKC activation. Wild-type E14TG2a.4 ESCs and RST613 single cell suspensions formed embryoid bodies (EBs) by forced aggregation into AggreWell 400 inserts (Stem Cell Technologies, Vancouver, CA) [24]. After 24 hours of culture in the wells, aggregates were transferred into Petri dishes containing 10 ml of serum-free N2B27 media, which consisted of DMEM/F12 (50/50) medium (Gibco, Grand Island, NY) supplemented with N2 (Gibco), 25 µg/L bovine serum albumin (BSA), 100 U/mL penicillin, 100 µg/mL streptomycin, and 0.25 µg/mL amphotericin (Mediatech), 2 mM l-glutamine (Mediatech), all combined 1∶1 with Neurobasal medium (Gibco) supplemented with B27 (Gibco) [25]. Aggregates were maintained on a rotary orbital shaker at 40 rpm. Mesoderm induction was attained by additional supplementation with 10 ng/mL BMP-4 (R&D Systems, Minneapolis, MN) during days 5–14 of culture. On day 14, EBs were treated with vehicle (0.01% ethanol) or 10^−8^ M 1α,25(OH)_2_D_3_ for 9 minutes. EBs were then lysed in RIPA buffer, and PKC activity was measured and normalized to total protein content as described previously [26].

The ESCs were injected into C57BL/6 blastocysts to create chimeric mice, which were bred with C57BL/6 mice to generate heterozygous *Pdia3* ^+/−^ deficient mice. The *Pdia3* ^+/−^ offspring were then intercrossed. Mice were genotyped by PCR amplification of tail DNA using prime pairs of a–b and a–c (Fig. 1A). Sequences of the primer sets used for PCR are listed in Table S1. The mice were weaned at 21 days of age, housed in a barrier facility with a 12-h light-dark cycle, and maintained on standard chow.

### Examination of mice embryos

To obtain embryos from different stages, *Pdia3* ^+/−^ heterozygotes were intercrossed to produce timed pregnancies. At 1 p.m., we checked the vaginal plug and considered it as E0.5. Embryos were isolated at different days of the pregnancy and stained with arcridine orange (Sigma, USA) in PBS for 30 min at 37°C. After extensive washes with PBS, the embryos were examined by stereoconfocal microscopy. For detection of transgenic β-galactosidase activity, the embryos were subjected to X-gal (Sigma, USA) staining according to the manufacturer’s protocol. After examining the embryos microscopically, RNA, DNA and protein were extracted from them using Trizol according to the manufacturer’s instruction (Invitrogen, USA). DNA was subjected to genotyping using primers described above, RNA was reverse transcribed into cDNA and amplified using primers against *Pdia3*, ribosomal protein s18 (*S18)* and neomycion (*Neo*). Sequences of the primer sets used for PCR are listed in Table S1.

Proteins were solubilized in 2% diethylamine after TRIzol extraction and subjected to 4–20% SDS/PAGE [27]. Blots were incubated with antibodies against PDIA3 (Alpha Diagnostic International, San Antonio, Texas, USA) or glyceraldehyde-3-phosphate dehydrogenase (GAPDH) (Millipore, Billerica, MA) and imaged with VersaDoc imaging system (Bio-Rad, USA) as described previously [12].

### Gross examination of adult mice

The wild type and heterozygous mice at 5, 10, 15 and 30-weeks-old were euthanized with CO_2_ inhalation following the IACUC protocol. The body length was measured as the distance between the nose and the end of the tail using a conventional ruler and the bodies were weighed. Multiple organs including liver, kidney, and heart were carefully dissected and weighed. The tibias on both sides were dissected, the surrounding skin and muscle were removed and the length of the tibias was measured using a caliper. Expression of *Pdia3* in the mice was measured in liver tissue to verify that the heterozygotes had reduced levels of this mRNA.

### µCT imaging and analysis

High-resolution µCT images of the left tibia of 5, 10, 15 and 30-week-old *Pdia3* ^+/+^ and *Pdia3* ^+/−^ mice (n = 6) were obtained using a Scanco VivaCT40 (Scanco Medical, Basserdorf, Switzerland) with a voxel resolution of 12 µm. Specific regions of analysis included the proximal tibial epiphysis, metaphysis, mid-diaphysis and distal growth plate. Standard Scanco software was used to determine microarchitecture morphology [16]. The growth plate was isolated with user-guided contours and evaluated at a threshold corresponding to 250 mg hydroxyapatite/cm^3^. Parameters derived at the tibial epiphysis and metaphysis included tissue volume (mm^3^), bone volume (mm^3^), bone volume/tissue volume (BV/TV %), trabecular number (#/mm), thickness (mm), and spacing (mm). At the cortical tibia mid-diaphysis, mean cortical area (mm^2^) and cortical thickness were determined through the scanned volume of bone.

Tethers are bony structures in the growth plate linking the epiphysis and metaphysis and are present in mice, rats, dogs, pigs, and humans [22], [28]. They have been linked to changing growth plate morphology and fusion, and to providing tissue stability. Our previous study extensively characterized the tether structure in mouse growth plates and demonstrated its correlation with functional *nVdr* [22], [28]. Therefore, in this study, we adopted this parameter to characterize Pdia3 heterozygous growth plates in addition to other conventional measurements including growth plate cartilage volume and properties of the hypertrophic zone. To qualitatively assess tissue morphology and tether distribution, 3D reconstructions of the growth plate were used to create 3D color images to visualize growth plate morphology and 3D tether maps to visualize formation within the growth center and along the surface [28]. Parameters derived in growth plate including growth plate volume (mm^3^) and tether volume/growth center volume.

### Histology and histomorphometric analysis

For histology and histomorphometric analysis, the left tibiae were fixed overnight in 4% paraformaldehyde. After rinsing in PBS, the samples were subjected to decalcification by incubating with cal-EX solution (Fisher Scientific Inc., USA) for 2 weeks. Decalcified samples were then embedded in paraffin and sectioned at 5-µm thickness. After deparaffinization, the sections were then subjected to trichrome staining. Images were captured using a Nikon Eclipse E100 microscope (Nikon Instruments Inc., Melville, NY). The thickness of the proximal tibial growth plate and the number of hypertrophic cells were then measured using Image Pro software.

### In vitro osteogenic differentiation of bone marrow progenitor cells isolated from wild type and heterozygous mice

Phenotypic changes were evident in the tibias at 15 weeks of age (see Results below), suggesting that changes at the cellular level had occurred before that time. Accordingly, bone marrow stromal cells (BMSCs) were isolated from 10-week-old wild type and heterozygous mice tibiae as described previously [16]. Briefly, tibias from *Pdia3* ^+/+^ and *Pdia3* ^+/−^ mice (n = 6 per group) were flushed using a 21-gauge needle attached to a 10 mL syringe filled with DMEM (GIBCO BRL, Gaithersburg, MD). Cells were filtered through a cell strainer with 70-micron nylon mesh (BD Bioscience, Bedford, MA) and then one million cells were plated in each well of a 12-well plate and cultured in mesenchymal stem cell growth media (α-MEM containing 10% FBS) at 37°C with 5% humidified CO_2_. The media were changed after 24 hours to remove non-attached cells and replaced with osteogenic medium (α-MEM containing 10% FBS, 0.2 mM dexamethasone, 10 mM β-glycerol phosphate and 50 µg/mL ascorbic acid). Media were replaced every 2 days for up to 28 days of culture. For measurement of osteoblast gene expression, cultures were harvested at day 14 and day 28. RNA was extracted using TRIzol (Invitrogen) and reverse-transcribed into cDNA using the Omniscript RT kit (Qiagen, Valencia, CA) according to the manufacturer’s directions. Real-time PCR was performed using SYBR Green SuperMix 170–8882 (Bio-Rad) for collagen type I (*Col1*), alkaline phosphatase (*Alp*), Runt-related transcription factor 2 (*RunX2*), bone sialoprotein (*Bsp*), osteopontin (*Opn*), and osteoprotegerin (*Opg*) and normalized to the level of housekeeping gene ribosomal protein s 18 (*S18*). The real-time PCR primers are listed in Table S2. Alkaline phosphatase activity was measured at day 28 and normalized to protein content [12]. The level of osteocalcin in the media at day 28 was measured using a mouse osteocalcin ELISA kit (Biomedical Technologies, Inc.) according to manufacturer’s protocol and normalized to DNA content. To assess mineralization, the 28-day cultures were fixed in 10% formalin; alizarin red and von Kossa staining were performed, and the positive staining area was quantified using ImageJ software [16].

### Statistical analysis

All measurements were collected with N = 6 independent determinations per data point. The data were expressed as mean ± standard error and were subjected to independent Student’s *t* tests. All statistical analyses were carried out using GraphPad 5.0 (GraphPad Software, Inc., La Jolla, CA). P<0.05 were considered as statistically significant.

## Results

### Generation of pdia3 deficient mice

The functional disruption of the *Pdia3* gene was confirmed by the loss of response to 1α,25(OH)_2_D_3_ induced rapid PKC activation in *Pdia3* ^−/−^ RST613 ESCs. While 1α,25(OH)_2_D_3_ treatment of wild type ESCs for 9 min resulted in a 4.9 fold increase in PKC activity compared to vehicle treatment (P<0.05), no significant PKC activation was observed in *Pdia3* ^−/−^ RST613 ESCs (Fig. 1B). These RST613 ESCs were then used to generate *Pdia3* knockout mice.

Global disruption of the *Pdia3* gene resulted in embryonic lethality. To determine the time of embryonic lethality, embryos were collected from heterozygous intercrosses at different days of gestation. No *Pdia3* ^−/−^ embryos were found after embryonic day 12.5. At E10.5, 2 out of 25 embryos were detected as *Pdia3* ^−/−^ by PCR analysis (Fig. 1C). The absence of *Pdia3* mRNA (Fig. 1D) and protein (Fig. 1E), and the presence of β-galactosidase activity (Fig. 1F) were confirmed by RT-PCR, western blot, and X-gal staining, respectively.

**Figure 2:**
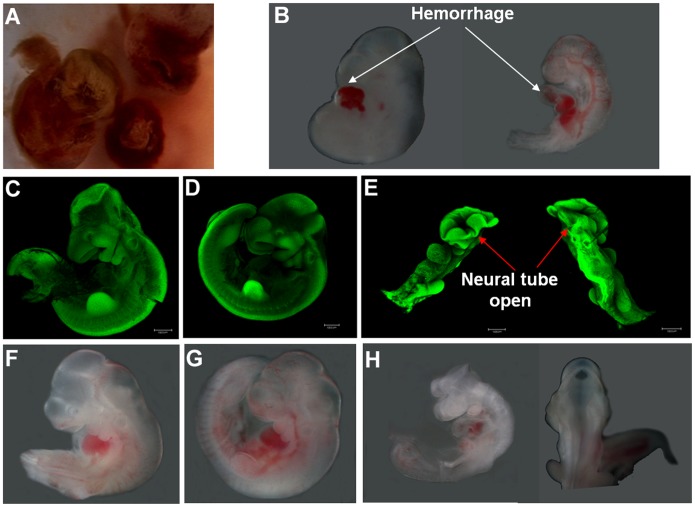
Disruption of pdia3 gene resulted in malformation during embryogenesis. The partially absorbed embryos were observed at E12.5 (A). E10.5 embryos were dissected and observed under stereomicroscopy (B, F–H) and confocal microscopy after acridine orange staining (C–E). The wild type embryos (C, F) and heterozygous embryos (D, G) were similar in size and larger than homozygous embryos. One homozygous embryo displayed hemorrhage around the heart area (B) and another one showed open neural tube (E, H). Scale bar = 1000 µm.

Reabsorption of dead embryos in utero was observed (Fig. 2A). Further stereo imaging analysis of E10.5 embryos showed that the *Pdia3* ^+/+^ (Fig. 2C, F) and pdia3^+/−^ embryos (Fig. 2D, G) were similar in size and morphology, while the two Pdia3^−/−^ embryos at best were 2/3 of the *Pdia3* ^+/+^ embryo size and displayed hemorrhage around heart region (Fig. 2B) or had an open neural tube (Fig. 2E, H).

**Figure 3:**
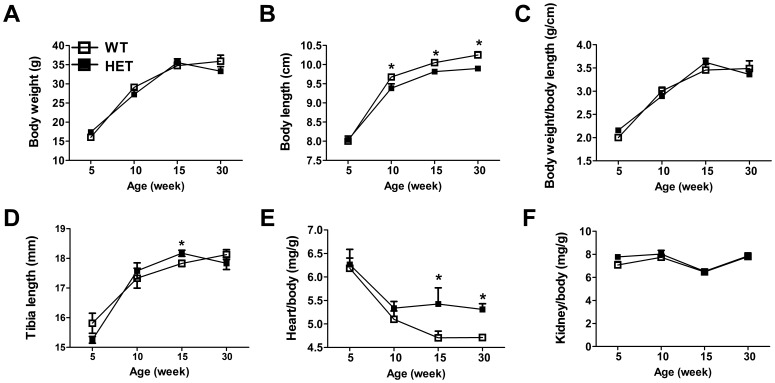
Gross analyses of pdia3 wild type and heterozygous mice. The tissues from wild type and heterozygous mice were harvested at 5, 10, 15, and 30 weeks of age. The lengths of the body and tibia as well as the weight of the body and organs were then measured. N = 6, *P<0.05 *vs*. wild type.

Heterozygous mice were fertile, exhibited a normal life span and did not show prominent pathological alterations. Genotype analysis of 500 offspring from *Pdia3* ^+/−^ intercrosses revealed 33% were Pdia3^+/+^ wild type, 67% were *Pdia3* ^+/−^ heterozygotes, and none were *Pdia3* ^−/−^ homozygous. Gross anatomy analysis (Fig. 3) showed that *Pdia3* ^+/−^ mice had short body lengths (week 10, 15 and 30) but long tibias (week 15), and larger heart/body ratios (week 15 and 30) in comparison to wild type mice. No significant differences in body weight, body weight/body length, and kidney/body ratio were observed.

### Expression of 1,25(OH)2D3 receptors in pdia3^+/−^ mice during the course of development

**Figure 4:**
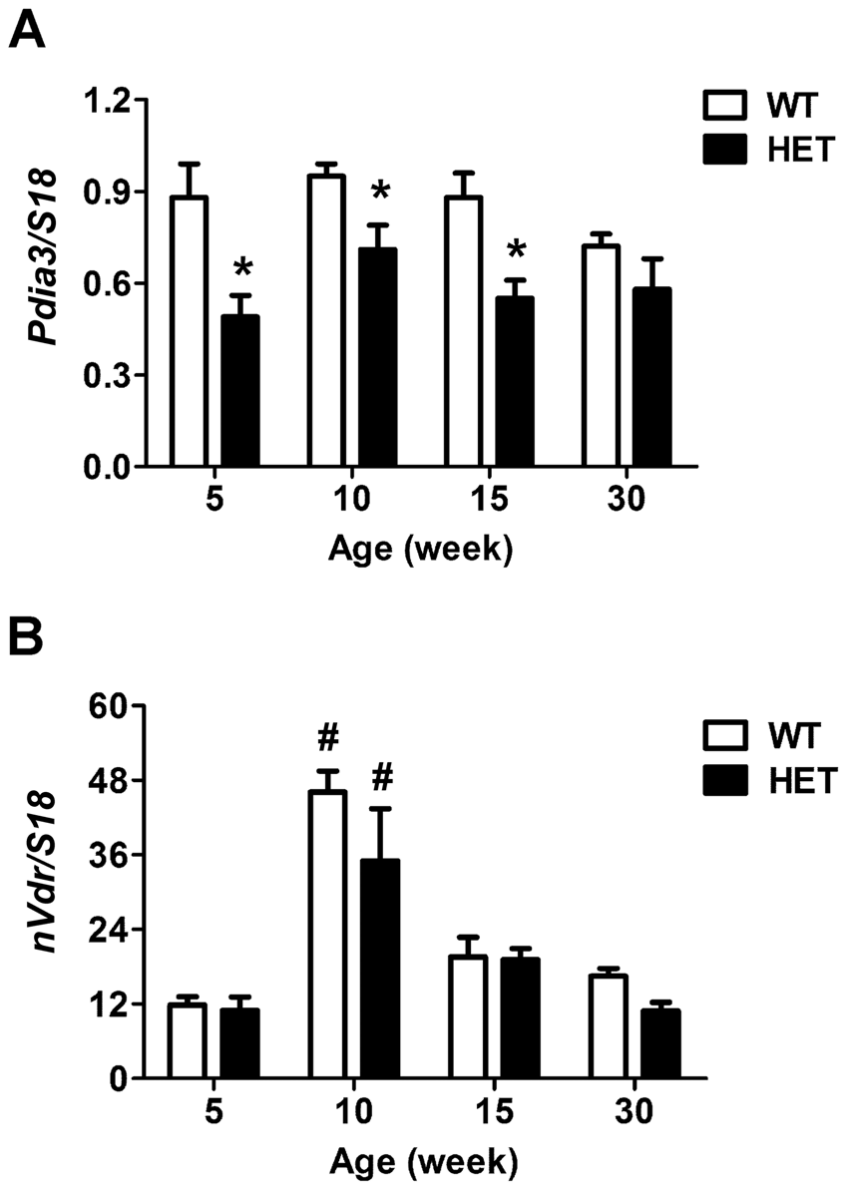
Gene expression for two types of 1,25(OH)_2_D_3_ receptors in wild type and heterozygous mice. RNA was isolated from the livers of wild type and heterozygous mice at 5, 10, 15, and 30-weeks of age. The gene expression of *Pdia3* (A) and *nVdr* (B) were analyzed by real-time PCR and normalized to *S18*. N = 6, *P<0.05 *vs*. wild type, ^#^ *P*<0.05 vs. week 5.

Real-time PCR analysis of liver tissue (Fig. 4) showed that the expression of *Pdia3* in heterozygous mice was 30–50% lower than that of wild type mice through week 15 but by week 30, differences in *Pdia3* expression between wild type mice and heterozygous mice were no longer evident. In contrast, *nVdr* expression in the liver was similar between wild type and heterozygous mice at all observation time points. *nVdr* expression increased at week 10 then decreased to the basal level (*P*<0.05) in both groups of mice.

### Growth plate abnormality in *pdia3^+/−^* mice

**Figure 5:**
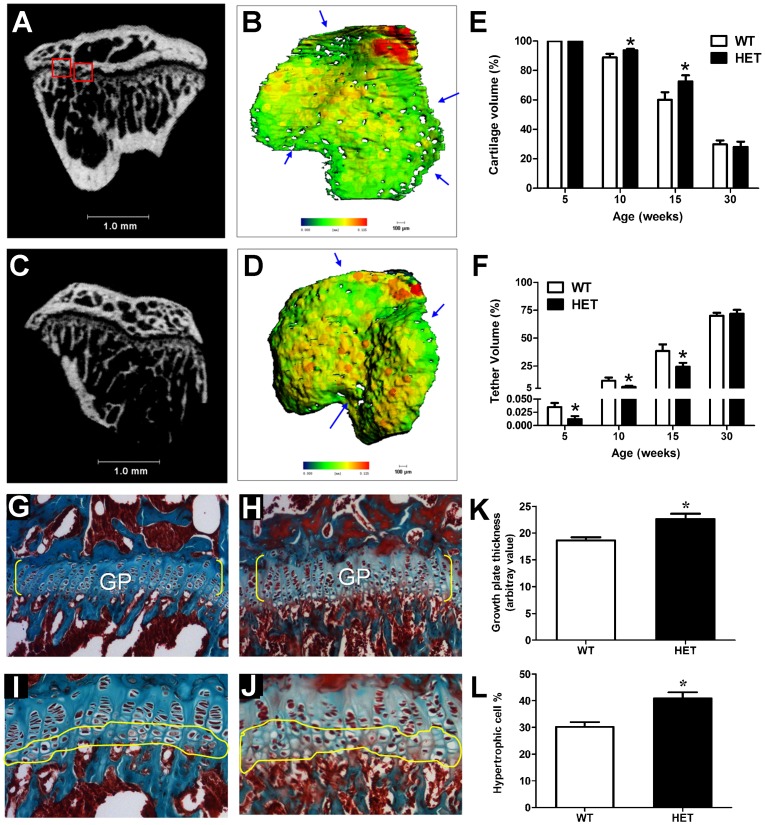
µCT and histological analyses of growth plates from *Pdia3* wild type and heterozygous mice. The left tibial growth plates were harvested at 5, 10, 15 and 30 weeks of age. Representative 2D cross section of proximal left tibia (A, C) and 3D reconstruction images of the growth plate (B, D) of 15-week of old wild type (A, B) and heterozygous mice (C, D). Tethers are indicated by a red box in 2D sections or an arrow in 3D images. The morphometric analyses of µCT scans showing cartilage volume (E) and tether volume (F). Trichrome staining of a cross section of the proximal left tibias of 10-week old wild type (G, I) and heterozygous mice (H, J). Histomorphometric analysis of growth plate thickness (K) and the percentage of hypertrophic cells per growth plate section (L). White bar represents wild type mice and black bar represents heterozygotes. N = 6, *P<0.05 *vs*. wild type.

The growth plates of 10-week-old wild type mice contained a number of tethers that displayed as bony bridge structures crossing the growth plate and connecting the epiphysis and metaphysis in 2D µCT images (Fig. 5A) or hole-like structures in 3D reconstruction images (Fig. 5B). However, these structures were barely observed in the growth plates of heterozygous mice (Fig. 5C, D). Quantitative analysis showed an age-dependent increase in tether volume in both types of animals but it was significantly lower in *Pdia3* ^+/−^ mice in comparison to wild type mice at week 5, 10 and 15 (Fig. 5F). In contrast, the cartilage volume of growth plates was significantly higher in *Pdia3* ^+/−^ mice compared to wild type animals at both week 10 and 15 (Fig. 5E). Histomorphometric analysis of histological sections also confirmed that the growth plates of 10-week-old heterozygotes were about 20% thicker and hypertrophic zones contained 12% more cells than those of wild type mice (Fig. 5G–L). Taken together, these results consistently and collectively suggested a decrease of mineralization of growth plate in *Pdia3* ^+/−^ mice.

### Bone phenotype in *pdia3^+/−^* mice

An age-dependent bone phenotype was observed in tibias of *Pdia3* ^+/−^ mice by µCT and morphometric analysis (Tables 1 and 2). At week 5, no significant difference in trabecular bone was found between wild type and heterozygous mice but at week 10, *Pdia3* ^+/−^ mice had a significantly lower number of trabeculae within the epiphysis and BV/TV within the metaphysis in comparison to wild type mice. At week 15, *Pdia3* ^+/−^ mice displayed significantly lower BV/TV and smaller trabecular thickness, but larger trabecular space within both the epiphysis and metaphysis compared to wild type. At week 30, no significant difference was observed within the epiphysis. In contrast, the *Pdia3* ^+/−^ mice exhibited significantly higher BV/TV within the metaphysis in comparison to wild type. Notably, no significant difference in cortical bone was found at all ages (data not shown).

**Table 1:** µCT analyses of tibia epiphyseal and trabecular bone phynotypes.

	BV/TV(%)	Trabeculae(#/µm)	Trab. Th.(µm)	Trab. Sp.(µm)
5 weeks				
* Pdia3* ^+/+^	58.3±2.4	14.4±0.8	0.060±0.004	0.071±0.003
* Pdia3* ^+/−^	56.6±2.3	14.0±0.7	0.059±0.003	0.074±0.003
10 weeks				
* Pdia3* ^+/+^	69.2±2.0	21.1±1.3	0.060±0.005	0.058±0.002
* Pdia3* ^+/−^	68.5±2.8	17.5±1.2*	0.062±0.007	0.058±0.008
15 weeks				
* Pdia3* ^+/+^	72.8±3.1	14.1±0.7	0.083±0.004	0.072±0.004
* Pdia3* ^+/−^	59.3±2.2*	15.2±1.7	0.068±0.004*	0.084±0.003*
30 weeks				
* Pdia3* ^+/+^	53.9±2.5	16.1±2.4	0.062±0.006	0.078±0.008
* Pdia3* ^+/−^	57.8±2.1	15.6±1.4	0.067±0.002	0.066±0.006

N = 6, *P<0.05 *vs*. wild type.

**Table 2:** µCT analyses of tibial metaphyseal and trabecular bone phynotypes.

	BV/TV(%)	Trabeculae(#/µm)	Trab. Th.(µm)	Trab. Sp.(µm)
5 weeks				
* Pdia3* ^+/+^	16.3±1.5	5.5±0.2	0.046±0.001	0.183±0.008
* Pdia3* ^+/−^	14.4±0.8	5.3±0.3	0.046±0.002	0.190±0.008
10 weeks				
* Pdia3* ^+/+^	14.4±1.8	6.3±0.7	0.050±0.002	0.115±0.031
* Pdia3* ^+/−^	12.3±2.3	6.6±0.3*	0.053±0.002	0.115±0.008
15 weeks				
* Pdia3* ^+/+^	27.4±1.3	5.8±0.2	0.058±0.002	0.152±0.005
* Pdia3* ^+/−^	19.6±1.4*	5.6±0.2	0.054±0.001*	0.170±0.006*
30 weeks				
* Pdia3* ^+/+^	6.9±0.5	4.2±0.1	0.046±0.002	0.239±0.008
* Pdia3* ^+/−^	10.3±1.8	4.4±0.2	0.049±0.002	0.228±0.013

N = 6, *P<0.05 *vs*. wild type.

### Impaired osteoblast differentiation potential of BMSCs isolated from *pdia3^+/−^* mice

**Figure 6:**
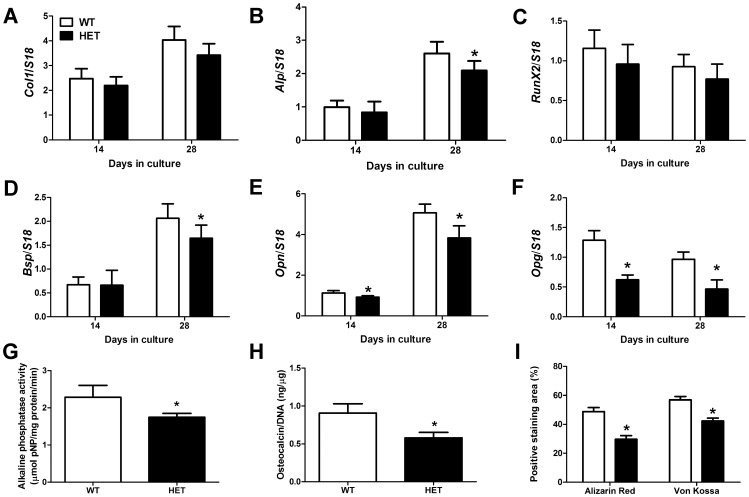
Reduced osteogenic differentiation capacity of bone marrow stem cells isolated from pdia3^+/−^ mice. The bone marrow stem cells (BMSCs) were isolated from wild type and heterozygous mice and cultured in osteogenic media for 14 and 28 days. The expression of collagen type I (*Col1*, A), alkaline phosphatase (*Alp*, B), Runt-related transcription factor 2 (*Runx2*, C), bone sialoprotein (*Bsp*, D), osteopontin (*Opn*, E) and osteoprotegerin (*Opg*, F) were evaluated by real-time PCR and normalized to housekeeping gene ribosomal protein s 18 (*S18*). Alkaline phosphatase activity (G) and the production of Osteocalcin (H) were measured in Day 28 cultures. Alizarin red and von Kossa staining were performed on Day 28 cultures to assess the mineralization and the positive staining areas were measured (I). White bar represents wild type culture and black bar represents heterozygous culture. N = 6, *P<0.05 *vs*. wild type.

Osteoblast differentiation of BMSCs was affected by *Pdia3* deficiency. Although the expression of col1 and runx2 was similar between the two types of cultures (P>0.05), the expression of several osteoblast markers including alp, bsp, opn and opg was significantly lower in *Pdia3* ^+/−^ cultures in comparison to wild type cultures at day 14 or day 28 (Fig. 6 A–F). In addition, heterozygous cells also displayed significantly lower alkaline phosphatase activity and produced less amounts of osteocalcin than wild type cells (Fig. 6 G, H). By the end of 28 days of osteogenic differentiation, the deposition of mineralized extracellular matrix was less in heterozygous cultures than in wild type cultures as indicated by smaller positive staining area of Alizarin red and von Kossa (Fig. 6I).

## Discussion

Due to embryonic lethality of global knockout of *Pdia3*, we developed mice with null mutation in one allele of the gene in order to study its role in skeletal development in post-fetal mice. Comparison of wild type and heterozygote mice showed that *Pdia3* deficiency results in mild skeletal manifestations at both growth plate and trabecular bone levels. The expansion of the growth plate was associated with an increased hypertrophic zone but decreased tether formation, which might contribute to the reduced trabecular bone volume [15], [28]. The impaired bone formation can also be attributed to reduced osteogenic potential of bone marrow cells in *Pdia3* ^+/−^ mice as evidenced by their lower mineralization capacity and reduced expression levels of osteoblast markers when cultured in osteogenic media. Changes in bone quantity may also be caused by altered bone resorption [16], which was not investigated in this study.

The skeletal manifestations in *Pdia3* ^+/−^ mice varied with post-natal age and on the skeletal region. Lower numbers of growth plate tethers in *Pdia3* ^+/−^ mice were first detected at week 5. The reduced number of tethers did not change with time, but growth plate volume was larger, the number of hypertrophic cells was greater and the number of trabeculae was reduced at week 10 in the *Pdia3* ^+/−^ mice. The differences in bone parameters between wild type mice and heterozygotes were most pronounced at week 15 in both epiphysis and metaphysis. To our surprise, while no significant differences were found in the growth plate and epiphyseal bone at week 30, *Pdia3* ^+/−^ mice displayed a higher metaphyseal bone volume in comparison to wild type mice. This observation was consistent with our previous study on the femoral metaphysis of 15-week-old *Pdia3* ^+/−^ mice, which showed significant increases in trabecular and cortical bone volume [26].

The mechanisms underlying the age- and location-dependent skeletal phenotypes of *Pdia3* ^+/−^ mice are still not clear. However, recent studies provide some clues that may contribute to the observations reported here. First, Pdia3 and nVDR might serve as a dominant 1α,25(OH)_2_D_3_ signaling mediator at different development stages. Others have shown that in chickens, 1α,25(OH)_2_D_3_ elicits rapid stimulation of phosphate and calcium transport in paralle with PKC activation in young birds but not adult birds, due to gradual decrease in the binding affinity of PDIA3 for 1,25(OH)_2_D_3_ [9]. In contrast, nVDR signaling remains either unchanged or increases with age [9]. We reported that PDIA3 is present in ESCs and mediates rapid 1α,25(OH)_2_D_3_-dependent PKC activation prior to expression of *nVdr* [29]. Moreover, *Pdia3* expression remains constant, whereas *nVdr* expression increases over time. Similarly, in the present study, *Pdia3* expression was constant over time but there was an increase in *nVdr* expression at week 10. The reduction in *nVdr* mRNA at later time points suggests that PDIA3 signaling may play a more dominant role in mediating the effects of 1α,25(OH)_2_D_3_ after week 10, which might explain why disruption of *Pdia3* led to the majority of bone phenotypes to occur in heterozygotes at week 15. Further examination of PDIA3 protein production in the growth plate and bone as well as functional disruption of 1α,25(OH)_2_D_3_ induced PKC activity in these *Pdia3* ^+/−^ cells are essential to elucidate the cellular mechanism of PDIA3’s action on skeletal development.

The differential influence of disruption of *Pdia3* on bones at different regions might also be due to their unique skeletal development process, geometrical and mechanical properties. The epiphysis is formed during embryonic bone formation, whereas the metaphysis is established post-fetally [16]. A progressive skeletal phenotype was also observed in *Cav-1* ^−/−^ mice from epiphysis to metaphysis [16]. Compared to the epiphysis, metaphyseal bone also has much larger contact surface area with bone marrow, which can regulate the bone formation through dynamic remodeling [30]. Moreover, femurs and tibias have distinct geometrical and mechanical properties [31], [32], which can also lead to their different phenotypic responses to PDIA3’s actions.

The skeletal phenotype of *Pdia3* ^+/−^ mice was similar but not identical to those of *nVdr* ^−/−^ mice and/or *Cav-1* ^−/−^ mice. Additionally, the extent of skeletal manifestations in *Pdia3* ^+/−^ mice was not as pronounced as in the other two models, which might be due to the partial deletion of *Pdia3* rather than complete ablation of *nVdr* or *Cav-1*, respectively. All of these animals have expanded hypertrophic cartilage. Both *nVdr* ^−/−^ and *Pdia3* ^+/−^ mice have reduced tether formation indicating of failure of calcification of cartilage [22], [28], whereas a delayed closure of the growth plate in *Cav-1* ^−/−^ mice is responsible for growth plate expansion [15], [16]. Both *nVdr* ^−/−^ and *Pdia3* ^+/−^ mice have reduced trabecular bone volume whereas *Cav-1* ^−/−^ mice exhibited increased bone formation [16], [22]. In terms of osteogenic potential, both osteoblasts from *nVdr* ^−/−^ mice and bone marrow cells from *Cav-1* ^−/−^ mice have demonstrated enhanced mineralized matrix formation and increased expression of osteoblast markers [16], [33]. In contrast, osteoprogenitor cells from bone marrow of *Pdia3* ^+/−^ mice showed decreased osteogenic potential.

While it is not surprising that these three mice models share some features in their skeletal phenotypes in view that nVDR, PDIA3, and CAV-1 are all involved in 1α,25(OH)_2_D_3_ signaling pathways and the potential cross-talk among them, their unique features indicate that these proteins might regulate skeletal development through mechanisms other than as 1α,25(OH)_2_D_3_ effectors. It has been suggested that CAV-1 can recruit β-catenin to caveolae and facilitate its subsequent nuclear translocation to activate the canonical Wnt pathway and regulate osteoblast differentiation and stem cell renewal [34], [35]. Our *in vitro* studies also show that silencing *nVdr* or *Pdia3* in osteoblast-like MC3T3-E1 cells leads to increased expression of BMP-2 and its inhibitor Noggin [13], which indicates that these two proteins might be involved in osteoblast commitment thorough regulation of the BMP-2 signaling pathway.

Of note, in addition to their skeletal phenotype, a greater heart size of *Pdia3* ^+/−^ mice was also observed after week 15, which indicates the potential role of PDIA3 in cardiovascular development. Interestingly, *nVdr* knockout and heterozygous mice also exhibit larger hearts than wild type mice as well as cellular hypertrophic changes in heart tissue at 12-months, which is directly due to ablation of nVDR signaling [36], [37]. Further studies of the cardiovascular system in PDIA3-deficient mice will be needed in order to reveal the mechanism involved.

In this study, no *Pdia3* knockout mice were obtained after birth confirming that global *Pdia3* knockout is embryonically lethal, as has been reported by others using different strategies for generating the null mutation [7], [38]. The *Pdia3* ^−/−^ embryo ratio (8%, 2 of 25) at E10.5 was far below the predicted ratio of 25% based on Mendelian distribution. The underrepresented *Pdia3* ^−/−^ embryos at this stage indicated the reabsorption of mal-developed *Pdia3* null embryos in the uterus and suggested that *Pdia3^−/−^* embryos died before E10.5. Although the two identified null embryos were morphologically abnormal, the exact onset and causes of embryonic lethality were not defined in this study. Others have reported that *Pdia3* ablation caused embryonic lethality at E13.5 [38]. Different from our knockout strategy with insertion of the gene trap vector in intron 1 of the *Pdia3* alleles, that study inserted the vector into intron 4 and obtained a longer truncated PDIA3 protein containing partial functional groups [38], which might explain the different embryonic lethality stages observed between these two studies.

In conclusion, our results provide for the first time the *in vivo* evidence that PDIA3 is critical for normal skeletal development in post-natal mice. Partial disruption of *Pdia3* leads to impaired bone formation and genetic ablation of *Pdia3* causes embryonic lethality. Because the *Pdia3*-deficiency in this animal model is not limited to cartilage or bone cells, it is not possible to define the exact mechanism by which PDIA3 controls skeletal development. In vitro studies examining the signaling pathways involved in PDIA3-dependent effects of 1α,25(OH)_2_D_3_ in growth plate chondrocytes and osteoblasts [39] support the hypothesis that downstream actions of the vitamin D metabolite on cell function are responsible for some of the observed phenotypic outcomes in the *Pdia3^+/−^* mice.

Development of conditional knockout mice with Pdia3 inactivation in cartilage or bone is essential to elucidate the direct role of PDIA3 in endochondral bone formation. Chondrogenesis precedes osteogenesis during embryonic development; thus, a bone-specific conditional knockout would not necessarily capture effects of *Pdia3* ablation on musculoskeletal outcomes. Moreover, endocrine actions will not be excluded from these conditional knockout systems. Mineral homeostasis is affected in chondrocyte-specific *nVdr*-null mice [40], but it is not known whether PDIA3-deficiency will also impact mineral metabolism. Therefore, levels of Ca^++^ and phosphate in the blood as well as analysis of hormones or other factors involved in bone or mineral homeostasis will be vital to address the systemic effects of PDIA3 in controlling skeletal development [41]. Additionally, *in vitro* studies using *Pdia3* knockout ESCs will be a useful model at cellular level for investigating the engagement of PDIA3-mediated 1,25(OH)_2_D_3_ signaling and its potential crosstalk with nVDR and CAV-1 signaling pathways in skeletal development. Finally, given the fact that PDIA3 can regulate skeletal development, it might serve as a target for musculoskeletal tissue engineering applications in the future.

## Supporting Information

Table S1 **Genotyping primer sequence.**
(DOCX)

Table S2 **Real-time PCR Primer Sequences.**
(DOCX)
