# Supplementary material for: Impaired Bone Formation in Pdia3 Deficient Mice
Source: PLoS One. 2014 Nov 18;9(11):e112708. doi: 10.1371/journal.pone.0112708 (PMC4236091; doi:10.1371/journal.pone.0112708)
Supplement: Table S1 — Genotyping primer sequence. (DOCX) [file pone.0112708.s001.docx]

**Table S1 Genotyping primer sequence**

| **Primer name** | **Primer sequence (5’→3’)** | **Accession number** |
| --- | --- | --- |
| a | AAGCCACCCTGAACTGTAGAG | NM_007952 |
| b | CATACAGTCCTCTTCACATCCATGC | NM_007952 |
| c | GCTATGGTTGGGATGTATCG | NM_007952 |
| *Neo* forward | GCTGCTCTGATGCCGCCGTGTTC | SCU46780 |
| *Neo* reverse | GCTGCTCTGATGCCGCCGTGTTC | SCU46780 |
