# Supplementary material for: Impaired Bone Formation in Pdia3 Deficient Mice
Source: PLoS One. 2014 Nov 18;9(11):e112708. doi: 10.1371/journal.pone.0112708 (PMC4236091; doi:10.1371/journal.pone.0112708)
Supplement: Table S2 — Real-time PCR Primer Sequences. (DOCX) [file pone.0112708.s002.docx]

**Table S2 Real-time PCR Primer Sequences**

| **Gene** | **Forward primer (5’→3’)** | **Reverse primer (5’→3’)** | **Accession number** |
| --- | --- | --- | --- |
| *Pdia3* | CCAATGATGTGCCTTCTC | TGTGCCTTCTTCTTCTTC | NM_007952 |
| *nVdr* | AGGCAGGCAGAAGAGATGAG | AGGGATGATGGGTAGGTTGTG | NM_00954 |
| *Col1* | GCATGGCCAAGAAGACATCC | CCTCGGGTTTCCACGTCTC | NM_007742 |
| *Alp* | GTGGGCATTGTGACTACC | GGTGGCATCTCGTTATCC | NM_007431 |
| *Runx2* | CCGCCACCACTCACTACC | GATAGGATGCTGACGAAGTACC | NM_009820 |
| *Bsp* | TGGAGACGGCGATAGTTC | GAGAGTGTGGAAAGTGTGG | NM_008318 |
| *Opn* | AACTCTTCCAAGCAATTCC | TCTCACAGACTCACCG | NM_009263 |
| *Opg* | CGCCAACATTTGCTTTCG | TGCTCCCTCCTTTCATCA | NM_008764 |
| *S18* | CTCTAGTGATCCCTGAGAAGTTCC | ACTCGCTCCACCTCATCCTC | NM_011296 |
